# Anakinra treatment in patients with systemic-onset juvenil idiopathic arthritis: “The Valencia Experience”

**DOI:** 10.1186/1546-0096-9-S1-P71

**Published:** 2011-09-14

**Authors:** I Marvillet, I Calvo Penadés, B López Montesinos, A Marco Puche

**Affiliations:** 1 Rheumatologic Unit, Department of Paediatrics, La Fe Hospital, Valencia, Spain

## Background

Treatment with Interleukin-1 (Il-1) receptor antagonist (anakinra) has been reported to be effective in some patients with systemic-onset juvenile idiopathic arthritis (SoJIA).

## Aim

To examine the safety and the efficacy of anakinra treatment in a regional cohort of SoJIA patients.

## Methods

We retrospectively reviewed the medical records of patients with SoJIA treated with anakinra (1-3 mg/kg/day) in our unit between December 2004 and july 2010. Anakinra’s effects were studied on several parameters including fever, rash, erythrocyte sedimentation rate, C-reactive protein levels, arthritis joints count, assessment of disease activity (by physician and parent/patient) and pain (by parent/patient). Resulting data were analysed to characterize therapies, clinical course and adverse events.

## Results

A total of 22 patients were included. Their mean age at the onset of treatment was 8,6 (1,8-15,6) years; disease duration was 2,4 (0-10,2) years. They had been follow up for 11-56 months. Fever and rash were resolved in 18 patients (82%) within the first 3 months. Active arthritis persisted at 3 months in 22% of patients, at 6 months in 14%, and at 12 months in 22%. Sixteen patients (70%), including 6 patients receiving anakinra as first-line therapy, attained a complete response. One patient stopped anakinra due to severe skin reaction and two patients due to infections: one severe pneumonia and one positive intradermoreaction. We observed 10 episodes of macrophage activation syndrome in 9 patients (40%), 8 episodes were present at the SoJIA diagnosis and 2 while receiving anakinra.

## Conclusion

In our experience, first-line therapy with anakinra in SoJIA patients was associated with an improved resolution of symptoms. These results justify further studies of Il-1 inhibition as first-line therapy in SoJIA patients.

